# Recent technological advances in correlative light and electron microscopy for the comprehensive analysis of neural circuits

**DOI:** 10.3389/fnana.2022.1061078

**Published:** 2022-11-30

**Authors:** Hirohide Iwasaki, Sotaro Ichinose, Yuki Tajika, Tohru Murakami

**Affiliations:** Department of Anatomy, Gunma University Graduate School of Medicine, Maebashi, Japan

**Keywords:** light microscopy, electron microscopy, CLEM, fluorescent nanoprobes, 3D reconstruction

## Abstract

Light microscopy (LM) covers a relatively wide area and is suitable for observing the entire neuronal network. However, resolution of LM is insufficient to identify synapses and determine whether neighboring neurons are connected *via* synapses. In contrast, the resolution of electron microscopy (EM) is sufficiently high to detect synapses and is useful for identifying neuronal connectivity; however, serial images cannot easily show the entire morphology of neurons, as EM covers a relatively narrow region. Thus, covering a large area requires a large dataset. Furthermore, the three-dimensional (3D) reconstruction of neurons by EM requires considerable time and effort, and the segmentation of neurons is laborious. Correlative light and electron microscopy (CLEM) is an approach for correlating images obtained *via* LM and EM. Because LM and EM are complementary in terms of compensating for their shortcomings, CLEM is a powerful technique for the comprehensive analysis of neural circuits. This review provides an overview of recent advances in CLEM tools and methods, particularly the fluorescent probes available for CLEM and near-infrared branding technique to match LM and EM images. We also discuss the challenges and limitations associated with contemporary CLEM technologies.

## Introduction

The brain consists of a highly complex network of neurons that transmit information primarily *via* synapses. Describing the wiring diagram of neurons in the brain is a major clue to understanding how the brain’s cognitive functions occur. From this perspective, a comprehensive analysis of neural circuits (the Connectome project) has been developed globally (Swanson and Lichtman, 2016). Because neurons extend a long projection (axon) to their target to form synapses, the observation of neural circuits should cover a wide area. Light microscopy (LM) covers a relatively wide area and is suitable for determining the entire morphology of neurons. However, the resolution of LM is approximately 250 nm in the lateral direction and 500 nm −1 μm in the axial direction, which is not sufficient to observe fine structures such as synapses and intracellular organelles. Super-resolution microscopies, such as stimulated emission depletion, stochastic optical reconstruction microscopy, and photoactivation localization microscopy, offer resolutions of 20–100 nm, which is still insufficient for observing fine organelle structures (Yamanaka et al., 2014).

The resolution of transmission electron microscopy (TEM) is approximately 0.1 nm, sufficient for observing synapses and organelles. TEM has been used to observe the fine structures of synapses, such as synaptic clefts and vesicles. Formerly, TEM was used for the three-dimensional (3D) reconstruction of neurons. However, 3D reconstruction by TEM requires significant skills, effort, and time to produce ultrathin serial sections of sample blocks using a diamond knife for collection on grids. Recently, the resolution of scanning electron microscopy (SEM) has improved, and SEM is now frequently used to observe neurons (Koga et al., 2021). Because there are informative review papers published recently about the technological advances of 3D reconstruction by SEM (Parajuli and Koike, 2021; Schifferer et al., 2021), we have not described it in this review. Although 3D reconstruction by SEM is a powerful technique for identifying the connectome, the total size of the datasets becomes gigantic and hard to handle if the large volume of neural tissue is covered only by electron microscopy (EM). Furthermore, the images obtained by EM are black and white, and “segmentation” is required to extract the neurons of interest. Although many machine learning-based algorithms have been developed for segmentation (Januszewski et al., 2018), fully automated segmentation of EM images is still difficult and requires verification by the human eye to avoid segmentation errors.

### Correlative light and electron microscopy

Correlative light and electron microscopy (CLEM) is an approach for correlating images obtained by light and EM. There are several workflows for CLEM, which are generally divided into two categories: pre-embedding and post-embedding. In pre-embedding CLEM, samples are observed by LM before the specimen is embedded in resin and cut into ultrathin sections for EM observation. On the other hand, in post-embedding CLEM, samples are observed by LM after sectioning. In most cases, the specimen is first observed by LM and then observed by EM. CLEM combining LM and 3D SEMs (FIB-SEM/SBF-SEM) can also be categorized as pre-embedding CLEM. For post-embedding CLEM, ultrathin serial sections can be stained with antibodies and observed by LM, as in array tomography (Micheva and Smith, 2007).

Correlative light and EM is a powerful approach for observing a wide range of neural circuits at the synaptic level. In particular, when the neurons of interest are labeled with fluorescent probes, the LM information may facilitate the fully automated segmentation of EM images. However, it is generally difficult to match the images obtained using LM and EM, as only the structures labeled with fluorescent probes are visible in the LM, while non-labeled structures are ignored. In contrast, all structures, including non-neuronal cells, such as glial cells and blood vessels, are visible in EM, making it difficult to extract specific neurons identified by LM from EM images. Another reason for the difficulty in matching LM and EM images is that the fluorescence of frequently used fluorescent proteins, such as GFP, is quenched during sample preparation for EM. In particular, treatment with osmium tetroxide (OsO_4_) and dehydration greatly reduces fluorescence. Hence, matching LM and EM images using fluorescence from ultrathin sections is difficult. However, as described below, several probes are available for both LM and EM. These probes can be classified into two categories as follows: (1) probes whose fluorescence can be observed after OsO_4_ treatment and dehydration, and (2) probes that generate reactive oxygen mainly by photoconversion and promote the coloration of 3,3′-diaminobenzidine (DAB).

### Fluorescent nanoparticles for correlative light and electron microscopy

As mentioned above, most fluorescent proteins used in cell biology are not tolerant to the treatments associated with EM sample preparation. However, there are some materials whose fluorescence is tolerant with EM sample preparation and can be observed both by LM and EM. In this section, we introduce four nanoparticle probes whose fluorescence is unaffected by OsO_4_ treatment and dehydration.

**FIGURE 1:**
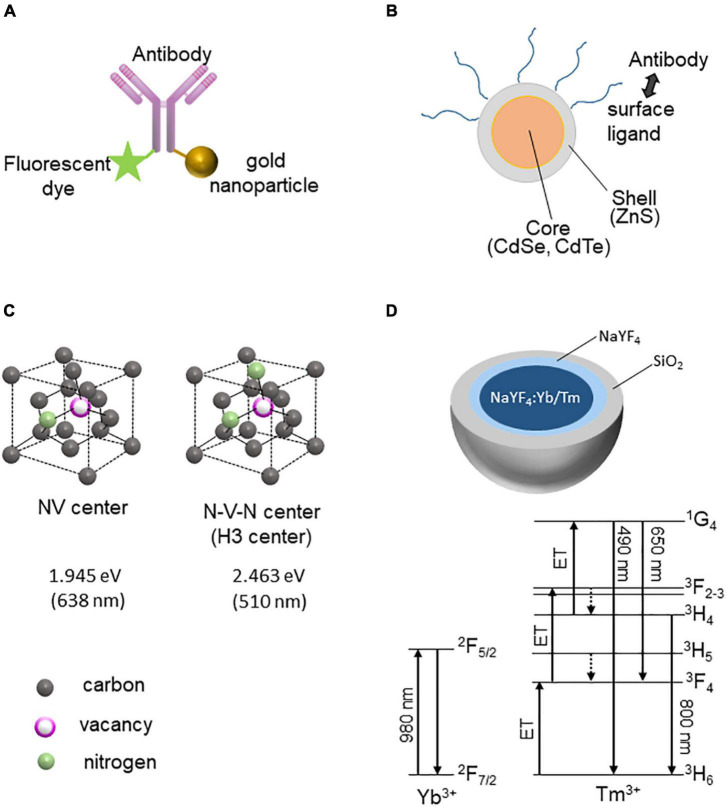
Fluorescent nanoprobes for CLEM. **(A)** FluoroNanogold structure: The gold nanoparticle and fluorescent dye are conjugated with the antibody (IgG or Fab fragment). **(B)** Quantum dot (Qdot) structure: Antibodies can be conjugated with Qdot *via* its surface ligands. **(C)** Crystallographic structures of NV and H3 (N–V–N) centers of fluoronanodiamonds. The energies and corresponding wavelengths of FNDs are shown (attributed to the zero-phonon lines). **(D)** Structure of upconversion nanoparticles with rare-earth ions (NaF_4_:Yb/Tm). The Jablonski diagrams of Yb^3+^ and Tm^3+^ are shown below.

FluoroNanogold is a complex of a fluorescent dye (Alexa Fluor) and gold particles bound to the Fab fragment of immunoglobulin G (or streptavidin) (Figure 1A; Powell et al., 1997; Robinson and Vandré, 1997). FluoroNanogold can label and visualize molecules of interest in neurons by attaching to either the Fab fragment of IgG or streptavidin. Several types of fluorescent dyes with different fluorescent spectra are available, enabling the distinction of multiple molecules in LM. The detection sensitivity of EM can be increased by silver enhancement.

Quantum dots (Qdots) are semiconductor nanocrystals containing cadmium mixed with selenium or tellurium (Figure 1B; Maysinger et al., 2015). The particle size of the nanocrystals determines the emission wavelength, and the fluorescence is directly observed by LM. Even a small number of probes in thin sections for TEM can be detected because their characteristic appearance means that Qdots can be easily distinguished from other substances in EM; thus, they can be used as probes for immunoelectron microscopy by labeling with antibodies (Killingsworth et al., 2012).

Fluorescent nanodiamonds (FNDs) contain color centers resulting from the crystal lattice defects in diamonds. Irradiation of high-energy particles, such as electrons and protons, causes displacement of carbon atoms and creates vacancies. Impurity atoms can be incorporated into these crystal lattice defects and interact with the electronic structures of the carbon crystal to generate a color center. Nitrogen atoms (N) are most often incorporated as impurities, and vacancies (V) trapped by nitrogen atoms form different color centers depending on the nitrogen state in the diamond. For example, vacancies adjacent to nitrogen atoms are called NV-centered diamonds, which emit orange/red fluorescence, whereas N-V-N (nitrogen-vacancy-nitrogen)-centered nanodiamonds emit green fluorescence (Figure 1C). The fluorescence of FNDs is very stable and does not fade (bleaching) or flicker (blinking), allowing long-term imaging. When an electron beam irradiates FNDs in SEM, in addition to secondary and backscattered electrons, it emits cathodoluminescence (CL) (Niioka et al., 2011). FNDs with different color centers emit light at different wavelengths. For example, FND with an NV center emits a red CL (λ∼ 620 nm), whereas FND with LuAG: Ce emits a green CL (λ∼510 nm). Multiple FNDs with different color centers can provide high-resolution multi-color SEM images (Glenn et al., 2012).

Upconversion nanoparticles (UCNPs) are unique probes due to their excitation and emission wavelengths. For most fluorescent probes, the wavelength of the excitation light is shorter than that of the fluorescence (Stokes shift). In contrast, UCNPs undergo a complex transition process by multi-photon multistep excitation, which results in an excitation wavelength that is longer than the fluorescence wavelength. For example, UCNPs doped with heavy rare-earth ions such as Tm^3+^ and Yb^3+^ emit blue light upon irradiation with near-infrared light at approximately 980 nm. Yb^3+^ is used to absorb near-infrared light at 980 nm, and the energy is transferred to Tm^3+^, which is responsible for the luminescence (Figure 1D; Chen et al., 2022). Instead of Tm^3+^, Ho^3+^, and Er^3+^ are also used to emit luminescence. Because most resins used in EM show autofluorescence, it is not easy to distinguish faint signals from resin autofluorescence; however, the special optical properties of UCNPs can be used to avoid the problem of autofluorescence. In addition, UCNPs emit fluorescence using infrared light (∼980 nm) as the excitation light and blue light, which is highly effective when combined with optogenetic techniques. In optogenetics, channelrhodopsin-2 (ChR2) can be expressed in neurons and stimulated with light at wavelengths of 450 and 475 nm to induce depolarization. However, because blue light is attenuated within the tissue, it cannot reach the deep region of the brain. Stimulating deep regions of the brain, such as the ventral tegmental area (VTA), requires that the optic fiber be inserted deep into the brain, which may cause damage to the brain. However, by injecting UCNPs with AAV encoding ChR2, neurons expressing ChR2 in the deep regions of the brain can be activated by irradiation with far-infrared light from the brain’s surface (Chen et al., 2018). Thus, UCNPs can control the function of neural circuits by optogenetics and are useful for observing neural circuits by CLEM.

Among the fluorescent nanoparticles listed above, FluoroNanogold has been commonly used for CLEM. The gold nanoparticles conjugated with fluorophores and other substances, such as antibodies or streptavidin, are commercially available. However, the organic dyes are relatively easy to quench and are affected by organic solvents used for EM sample preparation. The fluorescence of Qdot is more stable than that of organic dyes. Qdots with various emission wavelengths are available commercially, and the surface modification of Qdots is relatively easy. However, Qdots are toxic to cells and are not suitable for long-term live imaging. The fluorescence of FNDs is stable and does not fade (bleaching) or flicker (blinking) compared to Qdots and organic dyes, thus allowing for long-term imaging. FNDs are not toxic to cells and can be internalized into cells without detergent. For these reasons, FNDs are suitable for long-term live imaging and have been used to monitor intraneuronal traffic (Haziza et al., 2017). However, the color variation of FNDs is limited compared to Qdots and UCNPs; moreover, the conjugation of antibodies or other biological substances to the surface of FNDs is not easy because of their relatively large size. Because the fluorescence of UCNPs is stable and the toxicity of UCNPs is low, they are suitable for live imaging. Conjugation of biological substances is relatively easy, although the surface of UCNPs is highly charged. The disadvantage of UCNPs is that the optical filters specific to UCNPs are required for LM observation because of their unique excitation/emission properties.

### Fluorescent proteins as correlative light and electron microscopy probes

Most fluorescent proteins used in cell biology, such as GFP, are easily quenched by OsO_4_ fixation and dehydration. However, several proteins have fluorescence tolerance to OsO_4_ treatment and dehydration. For example, the fluorescence of mEos4a and mEos4b is not quenched by OsO_4_ treatment (Paez-Segala et al., 2015). However, to maintain fluorescence, commonly used hydrophobic resins, such as Epon, are not available for mEos4a and mEos4b. Instead, hydrophilic resins, such as lowycryl and LRWhite, should be used, and a high-pressure freezing method is necessary for embedding. However, high-pressure freezing requires special equipment and a limited sample size. Recently, mEosEM, which originated from mEos3.2 with some genetic modifications, was reportedly embedded in Epon by maintaining its fluorescence (Fu et al., 2020).

Tanida et al. (2020a,b) and Sanada et al. (2022) demonstrated the relative stability of mWasabi, Co-GFP variant 0, and mCherry2 fluorescence during OsO_4_ treatment and dehydration. Although the fluorescence of these proteins was quenched by OsO_4_ treatment, it was restored by subsequent treatment with TUK solution (FUJIFILM Wako Chemicals, #208-21161). Because these proteins have different fluorescence spectra, multi-color CLEM can be performed using these probes. However, the data shown in these reports are from experiments in which these proteins were overexpressed in cultured mammalian cells, such as HEK293 cells. Therefore, whether these fluorescent proteins can be used as CLEM probes when they are expressed in neurons remains unknown.

### Correlative light and electron microscopy probing by 3,3′-diaminobenzidine photoconversion

3,3′-diaminobenzidine (DAB) is oxidized by singlet oxygen species generated by light. Insoluble polymer of DAB is detectable not only by LM but also by EM as an electron-dense material. Therefore, DAB has been used for CLEM approach. The probes in this section are primarily protein-based and can be used as genetic tags. These probes should be introduced into the cells as transgenes. Attaching a tag sequence to specific organelles, such as mitochondria and the endoplasmic reticulum (ER), allows their specific labeling with DAB. However, DAB reactants may diffuse by approximately 100 nm and react with osmium tetroxide to form high electron density deposits. Therefore, if the probe diffuses throughout the cell, the deposits may hide the intracellular structures and interfere with observation.

APEX is an ascorbate peroxidase with approximately the same molecular size as GFP (27 kDa). The enzymatic activity of APEX is not affected by glutaraldehyde fixation (Martell et al., 2017). When cells expressing APEX are fixed with aldehyde and treated with DAB and H_2_O_2_, an insoluble complex is formed, which becomes visible in EM upon treatment with OsO_4_. APEX2 was derived from APEX to improve the efficiency of DAB polymer production. Because APEX2 is not fluorescent, fusion proteins of APEX2 with fluorescent proteins, such as GFP-APEX2, have been used as CLEM probes. When expressing APEX2-Venus-CAAX, a fusion protein of APEX2 with a membrane-targeted Venus, the neurons are sparsely labeled in the brain, and a single neuron can be observed both by LM and EM (Hirabayashi et al., 2018).

FlAsH/ReAsH is a biarsenical fluorophore that binds specifically to peptides containing amino acid sequences, such as CCXXCC, where X is an amino acid other than cysteine (Adams et al., 2002). Both FlAsH and ReAsH are highly permeable to cell membranes. When administered outside the cell, they can pass through the cell membrane, enter the cell, attach to the tetracystein sequence, and produce green (FlAsH) or red (ReAsH) fluorescence. Furthermore, these tags efficiently emit reactive oxygen species (ROS) to induce DAB photoconversion, which can be observed by EM. The tetracystein peptide is short and is easily introduced into the gene of interest as a tag; however, it is not easy to wash out the excess unbound FlAsH or ReAsH probe in tissues. Thus, the unbound FlAsH or ReAsH may cause high background staining (Hoffmann et al., 2010).

Mini singlet oxygen generator (miniSOG) is a fluorescent flavonoid protein derived from *Arabidopsis* phototropin 2. The fluorescence of miniSOG is caused by its flavin cofactor. MiniSOG efficiently generates singlet oxygen and forms a DAB polymer when irradiated with blue light (Shu et al., 2011). The molecular weight of miniSOG (15 kDa) is lower than that of GFP (27 kDa), indicating the advantage of miniSOG as a fluorescent tag. In particular, miniSOG can be used as a fluorescent tag when packaged into viral vectors because the packaging size in viral vectors is quite limited. However, miniSOG exhibits excitation under blue light (447 nm), which is toxic for live-cell imaging. When miniSOG is used to promote DAB complex formation, intense irradiation with blue light is needed; thus, the region to induce photoconversion is narrow.

### Matching light microscopy and electron microscopy images using fiducial markers

Characteristic structures in the samples can be used as fiducial markers to match LM and EM images. Two types of structures can be used as fiducial markers: the structure derived from the biological tissue and the structure created artificially by laser (laser ablation).

Blood vessels are commonly used as fiducial markers among the structures derived from biological tissues. Blood vessels appear dark in fluorescence microscopy images, owing to the lack of autofluorescence. The diversities in the running of blood vessels make them good fiducial markers for matching LM and EM images. 3D images of blood vessels can be reconstructed using the image stacks obtained by confocal laser microscopy and can be matched to the 3D reconstructed image by EM.

**FIGURE 2:**
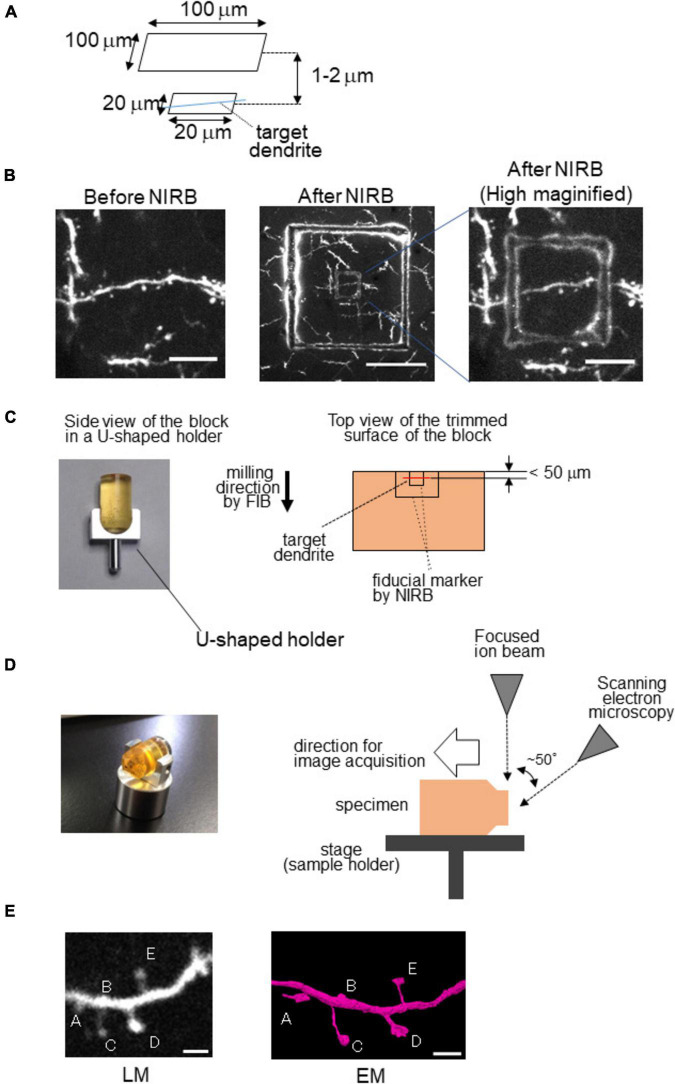
Near-infrared branding (NIRB) as a fiducial marker for CLEM. Example of NIRB marking in a fixed brain section for the 3D reconstruction of a target dendrite. **(A)** NIRB scheme for marking a target dendrite. **(B)** High-magnification images of the dendrite from the cerebral cortex of Thy1-EYFP-H mouse before (left) and after NIRB (middle, right). This mark can be easily visualized *via* light microscopy from its autofluorescence. Scale bars: 10 μm (left and middle) and 50 μm (right). **(C)** Block trimming of NIRB samples. (Left) Side view of the sample in a U-shaped holder (HV-8; Microstar, Japan) before trimming. (Right) Scheme for trimming the sample block for FIB-SEM imaging. The target dendrite should be located close to the edge of the block (<100 mm). **(D)** Observation of the sample block using FIB-SEM. (Left) Sample block after trimming in the U-shaped block holder (HV-8). The sample block is turned until the surface of the trimmed block is parallel to the focused ion beam (right) configuration of the dual-beam FIB/SEM and sample set. **(E)** (Left) Example of a two-photon microscopic image of a cortical dendrite *in vivo*. Scale bar = 2 μm. (Right) 3D reconstructed image of the target dendrite by EM. Modified from Takahashi-Nakazato et al. (2019) with permission.

Because both LM and EM can observe scars caused by laser ablation, they can be good fiducial markers. However, artificial fiducial markers are essential if there are no characteristic blood vessels in the vicinity of the neurons of interest. The near-infrared branding (NIRB) method is useful for marking the region of interest in the tissue by laser ablation (Maco et al., 2013). For NIRB, two-photon microscopy is useful for making a fiducial marker as the laser is focused within a limited space close to the focal point, especially in the axial direction. Thus, fiducial markers are limited in depth, which makes it easy to determine the target structure of interest, such as dendritic spines, which have been monitored for long periods by *in vivo* imaging (Figures 2A,B,E). While identifying the target dendrite at low magnification, it is useful to create an additional large square frame 1–2 μm above the small square frame at the same depth as the target dendrite (Figures 2A,B).

To make a fiducial marker using a laser, tissue should be sliced to 50–100 μm thickness using a vibratome before irradiation by laser. The laser should be used at a high excitation power (100–120 mW) to produce scars by laser ablation. Subsequently, the specimens are subjected to EM. For 3D reconstruction, FIB-SEM is a high-throughput, automated tool that is appropriate for the reconstruction of the target structure within a limited volume, such as dendrites and spines, because it uses a gallium ion beam to mill the surface of the block, the thickness of which can be accurately controlled. In addition, FIB-SEM images have a high axial resolution (∼10 nm) compared to images obtained *via* TEM or SBF-SEM (∼40 nm), which is also advantageous for revealing the fine structure of the target. However, it is challenging to obtain the target structure from a 3D reconstructed image using FIB-SEM, as sample preparation requires a relatively complicated process (Maco et al., 2014) because the FIB trims the sample block perpendicular to the surface of the block, while SEM scans the surface generated by the FIB from an angle, making it challenging to identify the target structure. However, to overcome this problem, a U-shaped sample holder (HV-8, Microstar, Japan) is useful to save time and effort (Figure 2C; Takahashi-Nakazato et al., 2019). After trimming the surface of the sample block until the fiducial markers appear on the surface of the block, the sample block is detached from the holder, and the direction whose surface was parallel to the FIB beam is changed (Figure 2D).

### Fixation protocols and correlative light and electron microscopy

Fixation is a crucial step in CLEM. In chemical fixation, which is frequently used to prepare specimens for EM, chemical cross-linking by aldehydes causes the quenching of fluorescent proteins. Fixation *via* freezing has long been used to avoid this problem. Cryofixation by high-pressure freezing is also frequently used; the Tokuyasu method is particularly useful for preserving sample structure and antigenicity and is suitable for immunoelectron microscopy (Tokuyasu, 1973). In contrast, cryofixation requires specialized equipment and has sample size limitations. Moreover, cryofixation does not apply to DAB labeling using APEX2, as dehydration after freeze displacement and *en bloc* staining with heavy metals are also limited in cryofixation, making it impossible to prepare samples for observation by SBF-SEM. To compensate for these disadvantages, the CryoChem method, which utilizes the advantages of both chemical and cryofixation, was recently developed (Tsang et al., 2018). In CryoChem methods, samples are first fixed by high-pressure freezing. After substitution in acetone solution with glutaraldehyde, uranyl acetate, methanol, and water, the samples are rehydrated gradually to increase the concentration of water to perform DAB-labeling or for observation *via* fluorescent microscopy. Subsequently, the samples are stained with osmium, rehydrated, embedded in the resin, and observed *via* EM. Using CryoChem methods, ultrastructures are preserved well, and samples can be used for DAB staining or fluorescence imaging. However, CryoChem also requires special equipment and has sample size limitations.

## Conclusion and perspectives

This review summarized various CLEM approaches and tools. The development of fluorescent probes (nanoparticles and proteins) for CLEM has been remarkable. However, each probe has its advantages and disadvantages, which require further improvement.

In addition to the development of CLEM methods, the combination of CLEM with other technologies may increase its importance. For example, DNA barcoding can distinguish each neuron thoroughly and provide precise information about neurons in neural circuits (Zador et al., 2012). Another promising approach is the combination of CLEM with X-ray holographic nano-tomography (XNH) (Kuan et al., 2020). The pixel size of XNH is 10–100 nm, and the field of view is 20–200 μm, which can cover the gap between LM and EM. Furthermore, XNH is a non-destructive approach, and the image can be acquired after embedding in resin for EM. Although XNH requires high-energy X-rays (>10 keV) and should be performed using a synchrotron, it can bridge the gap between LM and EM and will be useful for connectomics approaches.

## Author contributions

HI wrote the draft. All authors edited and finalized the manuscript and designed, modified the figures, contributed to the manuscript, and approved the submitted version.
